# Substance Use and Adherence to Antiretroviral Therapy among People Living with HIV in the United States

**DOI:** 10.3390/tropicalmed7110349

**Published:** 2022-11-04

**Authors:** Sarahmona Przybyla, Rebecca L. Ashare, Loriann Cioffi, Isabella Plotnik, Jonathan Shuter, Elizabeth K. Seng, Andrea H. Weinberger

**Affiliations:** 1 Department of Community Health and Health Behavior, State University of New York, Buffalo, NY 14214, USA; 2 Department of Psychology, State University of New York, Buffalo, NY 14214, USA; 3 Ferkauf Graduate School of Psychology, Yeshiva University, Bronx, NY 10461, USA; 4 Department of Medicine, Albert Einstein College of Medicine, Bronx, NY 10461, USA; 5 Department of Epidemiology and Population Health, Albert Einstein College of Medicine, Bronx, NY 10461, USA; 6 AIDS Center and Division of Infectious Diseases, Montefiore Medical Center and the Albert Einstein College of Medicine, Bronx, NY 10461, USA

**Keywords:** substance use, antiretroviral therapy, adherence, HIV/AIDS, United States

## Abstract

People with HIV (PWH) report substance use at higher rates than HIV-uninfected individuals. The potential negative impact of single and polysubstance use on HIV treatment among diverse samples of PWH is underexplored. PWH were recruited from the Center for Positive Living at the Montefiore Medical Center (Bronx, NY, USA) from May 2017-April 2018 and completed a cross-sectional survey with measures of substance use, antiretroviral therapy (ART) use, and ART adherence. The overall sample included 237 PWH (54.1% Black, 42.2% female, median age 53 years). Approximately half of the sample reported any current substance use with 23.1% reporting single substance use and 21.4% reporting polysubstance use. Polysubstance use was more prevalent among those with current cigarette smoking relative to those with no current smoking and among females relative to males. Alcohol and cannabis were the most commonly reported polysubstance combination; however, a sizeable proportion of PWH reported other two, three, and four-substance groupings. Single and polysubstance use were associated with lower ART adherence. A thorough understanding of substance use patterns and related adherence challenges may aid with targeted public health interventions to improve HIV care cascade goals, including the integration of substance use prevention into HIV treatment and care settings.

## 1. Introduction

In the United States (U.S.), there are important target goals for halting the HIV epidemic in the Department of Health and Human Services’ HIV National Strategic Plan for the United States: A Roadmap to Ending the Epidemic [1]. One key strategy to assess progress in HIV care and treatment outcomes includes a specific focus on viral suppression among people living with HIV (PWH). While high antiretroviral therapy (ART) adherence is a key determinant of sustained viral suppression, less than two-thirds of diagnosed PWH in the U.S. achieve this benchmark [2] which is well short of the 95% goal to end the HIV epidemic. These initiatives also exist in New York State, with similar metrics for viral suppression goals [3]. Optimal ART adherence is essential for PWH as it is associated with lower rates of opportunistic infections, hospitalization, and mortality [4]. Given that virologic suppression is closely correlated with ART adherence [5] understanding impediments to optimal adherence is a public health priority.

Research has consistently demonstrated that substance use is higher among PWH than the general population [6]. For example, U.S.-based samples of PWH find cannabis use rates ranging from 25–38% [7] compared to 18% of the general population [8]. In addition, stimulant rates ranging from 5–15% [9,10] have been reported compared to 2% of the general population [8]. Effectively addressing substance use among PWH is part of a larger effort to improve HIV care continuum outcomes and end the epidemic.

Of critical importance is the potential negative impact of substance use on HIV treatment engagement and maintenance. Specifically, substance use is one of the most commonly reported correlates of inferior adherence [11]. There are robust and consistent findings on the association between alcohol and ART non-adherence [12,13]. Other substances have been associated with ART non-adherence including heroin [10] and cocaine [13]. In contrast, research findings on the relationship between cannabis and ART adherence are mixed with some studies demonstrating an association between cannabis and sub-optimal adherence and others finding no association [14,15]. Methamphetamine use may also be predictive of ART non-adherence although studies are limited in scope to those including mainly or exclusively sexual minority men [16,17]. 

A more recent and growing body of literature has examined concurrent use of multiple substances, often termed polysubstance use (PSU), and its association with medication adherence challenges among PWH [18,19]. The public health significance is considerable; among adults living with HIV in the U.S., polysubstance use rates range from 7–28% [9,20]. Consequently, defining PSU patterns may have value in understanding behavioral risk and protective factors associated with medication adherence among PWH. Avoiding the traditional silo approach of exploring substances individually and independently, a more holistic exploration of substance use may provide a more nuanced understanding of adherence challenges among PWH. 

However, gaps remain in our understanding of PSU and medication adherence specifically related to sample representation. First, most studies examining single or polysubstance use and, by extension, their relationships to ART adherence, have focused exclusively or primarily on sexual minority men [19] with limited representation of women [14,21]. Although HIV diagnoses among women are on the decline in the U.S., nearly 20% of incident cases occurred among women in 2020 [22]. In addition, men and women living with HIV often have differing HIV engagement experiences [23] and patterns of ART adherence [24,25]. For example, a retrospective cohort of 33,224 PWH found that women were less likely to be engaged in HIV medical care, prescribed ART, and virally suppressed than men [26]. Second, while some studies have explored cigarette smoking as an independent correlate of ART nonadherence [27,28] there is limited research examining either the prevalence of polysubstance use or the extent to which smoking affects the PSU-ART adherence relationship [29]. This absence is important given that the prevalence of tobacco use is approximately twice as high among PWH relative to the general population [30,31] and PWH who smoke experience excess mortality associated with smoking [32]. In addition, a recent meta-analysis found smoking to be significantly associated with sub-optimal ART adherence [33]. According to the Centers for Disease Control and Prevention, tobacco smoking is arguably the most significant threat to ART-related health gains [34].

The current study aims to: (1) Characterize the prevalence of single and polysubstance use among PWH by gender and cigarette smoking status; (2) Assess the relationship between substance use (i.e., no use, single use, PSU) and ART adherence; and (3) Describe single and PSU patterns among adherent versus nonadherent PWH.

## 2. Materials and Methods

### 2.1. Participants and Procedures

Study participants were PWH receiving treatment at the Center for Positive Living (CPL) at the Montefiore Medical Center (Bronx, NY, USA) from May 2017–April 2018. The CPL serves approximately 2600 PLH. Approximately two-thirds of the patient population has an AIDS diagnosis, 56% are male, and 39% are Black/African American. Individuals were recruited for study participation in the CPL waiting room. Inclusion criteria included: age 18 or older, reporting an HIV diagnosis, ability to speak and read in English, and ability to provide oral informed consent for study participation. Consent procedures and completion of an anonymous survey were conducted in private rooms. More details on study procedures have been described [35]. All procedures and related activities were approved by the Institutional Review Board at Albert Einstein College of Medicine, and all participants provided informed consent. Participants received a $20 Target gift card upon study completion.

### 2.2. Measures

#### 2.2.1. Sociodemographic Characteristics

Participants self-reported their age, gender, education level, marital status, sexual orientation, and race. 

#### 2.2.2. HIV Clinical Characteristics

Participants self-reported the year of HIV diagnosis, AIDS diagnosis, and current use of ART. 

#### 2.2.3. Smoking Status

Participants were asked to self-report their current cigarette smoking status and were categorized into one of two mutually exclusive smoking status groups: (1) having current smoking (i.e., “I currently smoke cigarettes”) or (2) having no current smoking (“I used to smoke cigarettes, but I do not smoke now” or “I never smoked cigarettes”).

#### 2.2.4. Substance Use Characteristics

Participants self-reported (yes/no) current use of each of the following substances: alcohol, cannabis, heroin, cocaine, methamphetamine, heroin, and hallucinogens. Participants were classified into one of two mutually exclusive groups for each of these substances: (1) having current use or (2) not having current use. Polysubstance use was defined as the current use of two or more of these substances (i.e., alcohol, cannabis, heroin, cocaine, methamphetamine, heroin, and hallucinogens). Tobacco use was not included in the definition of polysubstance use, consistent with prior work among PWH [18,36,37].

#### 2.2.5. ART Adherence

Of those who reported current ART use, ART adherence was assessed as any reported missed doses of ART in the last seven days consistent with prior studies examining ART adherence and substance use [36,38,39]. Participants who reported that they missed any doses of ART in the past seven days were classified as “non-adherent” while participants who reported that they did not miss any doses of ART in the past seven days were classified as “adherent.” This operationalization was chosen to capture clinically relevant non-adherence.

### 2.3. Statistical Analyses

We summarized the descriptive statistics for all independent and dependent variables. First, chi-square tests for categorical variables and *t*-tests for continuous variables were used to examine and compare HIV clinical characteristics (e.g., years since HIV diagnosis) and demographic characteristics (e.g., age) overall and by cigarette smoking status, gender, and ART adherence. Second, we conducted bivariate analyses using chi-square and unadjusted odds ratios to compare substance use categories: (a) no substance use, (b) single use and (c) polysubstance use by gender, smoking status, and ART adherence groups. Multivariate logistic regression was conducted to examine the relationship between different substance use categories (i.e., one substance, polysubstance) and ART adherence controlling for gender and cigarette smoking status. We also constructed models with other covariates including age, race, marital status, and AIDS diagnosis, adding each separately and another model will all covariates included. When comparing models, the changes in Bayesian Information Criterion (BIC) were all greater than four, suggesting positive support for the original model. We elected to use the more parsimonious model with variables central to our hypotheses. Results were considered statistically significant at a two-tailed alpha level of 0.05. All data analyses were conducted in STATA, version 17. 

## 3. Results

### 3.1. Participant Characteristics

Among the 445 patients approached, 147 declined to participate or failed to meet inclusion criteria. Of the 298 individuals who provided oral consent, 61 were missing data on key variables of interest (e.g., 36 PWH did not report current ART use), resulting in a final analytic sample of 237 participants (42.2% female, 47.3% current smoking status, 64.1% ART adherent). Participant characteristics are reported in Table 1. Our overall sample was primarily heterosexual (73.8%) and Black (54.1%). The median age was 53 (interquartile range: 45–58 years). With respect to HIV clinical characteristics in the full sample, the median years since HIV diagnosis was 21 (interquartile range: 15–26 years), and nearly half of the sample reported an AIDS diagnosis. Participants did not differ by cigarette smoking status (smoking, no smoking) on any HIV clinical or sociodemographic characteristics other than sexual orientation. Chi-square tests detected significant differences between females and males in sexual orientation and marital status. Females were more likely to report an AIDS diagnosis relative to their male counterparts. 

### 3.2. Substance Use Characteristics

**Figure 1:**
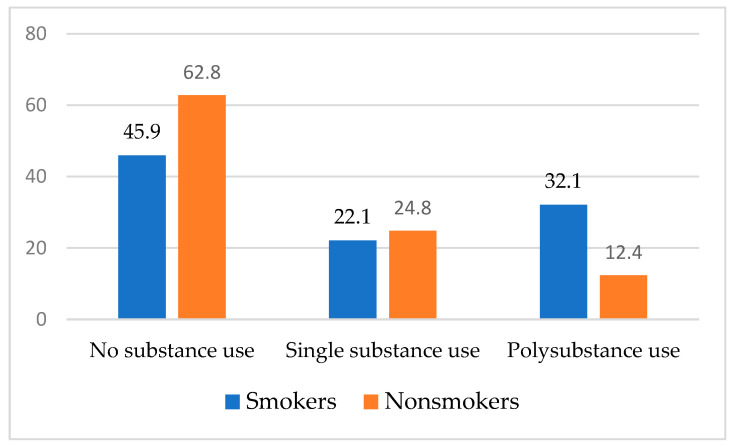
Comparison of Substance Use by Smoking Status.

**Figure 2:**
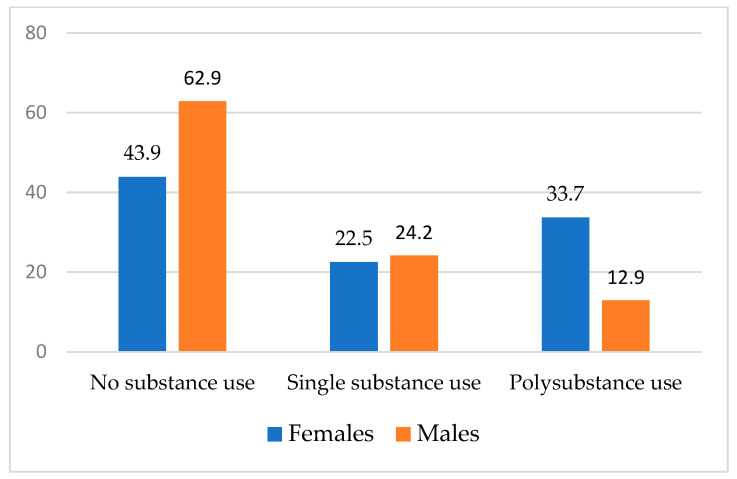
Comparison of Substance Use by Gender.

In the full sample, overall substance use was moderately high. Approximately half (44.5%) of participants reported any current use of at least one of the substances assessed (i.e., alcohol, cannabis, heroin, cocaine, methamphetamine, heroin, and hallucinogens). Approximately one-third (32.2%) reported current alcohol use, 26.1% reported current cannabis use, and 9.2% reported current cocaine use (data not shown). Across smoking status and gender, there were no differences in single substance use. Approximately one in five participants (21.4%) reported polysubstance use. Crude associations demonstrate that polysubstance use was more prevalent among those with current smoking relative to those without current smoking (OR = 1.99; 95% CI, 1.17–3.38) and among females relative to their male counterparts (OR = 2.17; 95% CI, 1.27–3.69), respectively (see Figure 1 and Figure 2).

### 3.3. Substance Use and ART Adherence

Overall, 152 participants (64.1%) were ART adherent by self-report in the previous seven days. Across groups, adherence was 72.4% (92/127) among those with no substance use, 54.7% (29/53) among those with single substance use, and 53.1% (26/49) among those with polysubstance use. In regression analyses controlling for smoking status and gender, substance use was associated with lower ART adherence (see Table 2). Compared to those participants who reported no substance use, single substance use [adjusted odds ratio (aOR) = 0.45, 95% CI 0.23–0.89] and polysubstance use [aOR = 0.46, 95% CI 0.22–0.96] were associated with decreased odds of being adherent. Neither smoking status nor gender was related to ART non-adherence. 

### 3.4. Single and Polysubstance Use by Adherence Status

**Figure 3:**
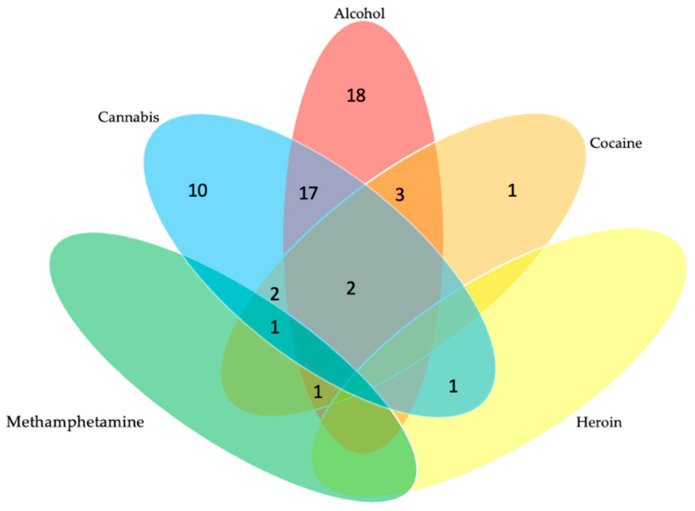
Substance Use Patterns Among a Sample of People with HIV who were ART Adherent (*n* = 55).

**Figure 4:**
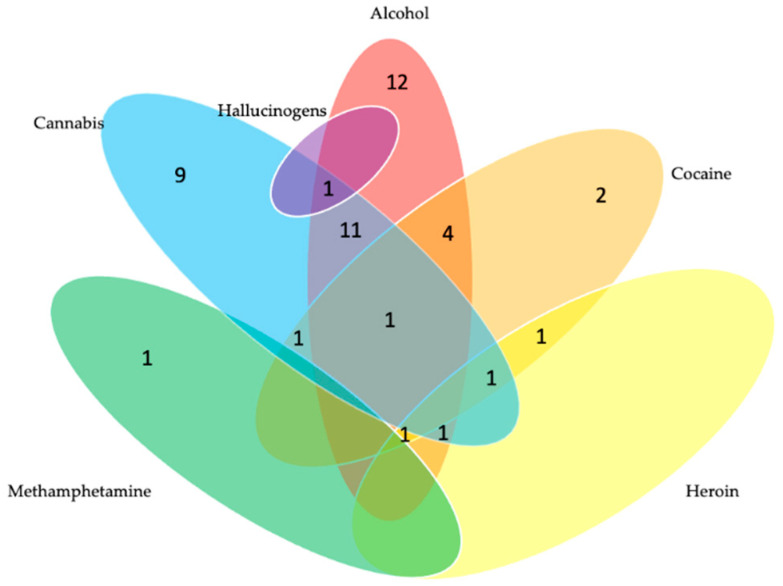
Substance Use Patterns Among a Sample of People with HIV who were ART Non-Adherent (*n* = 47). 1 participant endorsed use of methamphetamine, cannabis, and alcohol.

Figure 3 and Figure 4 illustrate the types and patterns of substance use among ART adherent and ART non-adherent study participants. Among the adherent group (Figure 3), 29 were single substance users (18 reported alcohol use, 10 reported cannabis use, and 1 reported cocaine use). In addition, 27 were polysubstance users (the most common combination was 17 individuals who reported alcohol and cannabis use). Among the non-adherent group (Figure 4), 24 reported single substance use (12 reported alcohol use, 9 reported cannabis use, 2 reported cocaine use, and 1 reported methamphetamine use). In addition, 22 reported polysubstance use (the most common combination was 11 individuals who reported alcohol and cannabis use). 

## 4. Discussion

In this cross-sectional analysis of PWH, we aimed to determine the extent to which substance use was associated with ART adherence in a sample comprised of racially and ethnically diverse, middle-aged PWH. Specifically, the study has several important findings. First, approximately half of participants reported any current substance use and one in five reported PSU, consistent with prior work with PWH [18,19]. While single substance use did not vary by smoking status or gender, we found that polysubstance use was more prevalent among women. Recent research has suggested that substance use in general and PSU in particular is largely understudied among women with HIV [21]. Further investigations should examine broader social and structural contexts (e.g., intimate partner violence, mental health comorbidities) to understand predictors and consequences of polysubstance use among women with HIV and the mechanisms underlying these patterns [40]. In addition, we found that polysubstance use was more prevalent among those reporting current cigarette use relative to those not reporting current cigarette use. While cigarette cessation can reduce both general and HIV-specific negative consequences of smoking [41], PWH are less likely to report quitting than people without HIV [42]. Further, in community and epidemiologic samples, substance use is associated with greater prevalence and lower quitting of cigarettes [43] and cigarette use is associated with greater substance use and poorer substance use treatment outcomes [44,45] although little is known about these relationships for PWH. The synergistic effects of nicotine, ART medications, and substance use warrant future studies on drug metabolism and HIV-related health outcomes. Given the high prevalence of cigarette smoking among PWH and its associated impacts on health and disease management [30,32,33], future mixed methods research can deepen our understanding of the relationship between smoking and ART adherence in diverse samples of PWH and the mechanisms by which substance use may affect HIV-related health outcomes among those who smoke cigarettes. 

Second, our findings demonstrate that PWH who use substances are more likely to be ART non-adherent than those who report no substance use. Our findings are consistent with prior work linking medication non-adherence with alcohol and recreational drug use among PWH [29,36,46,47,48,49]. These connections could likely result from impairment in cognitions impacting both unintentional nonadherence [50] such as memory lapses and intentional non-adherence [51,52] related to, for example, substance use-ART toxicity beliefs. These findings are clinically meaningful given that the use of substances such as methamphetamine, cocaine, and alcohol are associated with lower adherence. This can lead to HIV disease advancement through biological mechanisms such as lowering CD4+ T-cell count and promoting viral replication, thus aiding in the progression of HIV infection and community transmission [53]. Further research can extend our work on the polysubstance-ART adherence relationship by examining the extent to which these effects are additive or multiplicative [37].

Importantly, PSU was associated with ART non-adherence, contributing to the growing body of literature on PSU among PWH [19,37]. These findings suggest possible intervention strategies. As sub-optimal adherence is related to viral non-suppression, behavioral interventions that co-locate harm reduction programs with HIV treatment and care may make a meaningful impact on HIV health outcomes [54]. For example, the availability of substance use-related treatment programs among PWH have demonstrated effectiveness in improving ART adherence [55]. 

Third, the types of substances reported varied in our study sample. Among adherent and non-adherent PWH, we found that that alcohol and cannabis were most commonly reported polysubstance combination. However, we also found a sizeable proportion reporting other two and three-substance groupings. The current exploratory study extends previous work of single use and PSU among PWH by gaining an in-depth understanding of the types of current substance use patterns among PWH who use substances. The evidence of heterogeneity in PSU suggests that a one-size-fits-all approach is ill-suited to intervene on preventive strategies from a public health perspective. Greater appreciation for the distinct substance use arrays can allow for improved dialogue between patients and clinicians on targeted approaches to addressing associated adherence challenges [56].

Our study findings have important clinical and public health implications. As sub-optimal adherence often contributes to poor HIV disease management, worse health outcomes, and risk of virologic failure, findings reiterate the need for population-level approaches to understanding the complexities of substance use and engagement in HIV treatment and care among PWH. Optimal adherence is key to achieving federal goals of viral suppression in the HIV Care Continuum and is critical to ending the HIV epidemic [57]. Future research directions would benefit from more comprehensive measurement tools to assess substance use and medication adherence. For example, novel combinations of subjective and objective pharmacologic measures of substance use and ART would improve our knowledge on the physiological interactions between substance use and ART and extend to viral suppression outcomes. This work would also benefit from the inclusion of diverse study samples.

While our results highlight key points relevant for potential intervention, it is important to consider these findings in light of limitations. First, the cross-sectional nature of the data collection precludes us from inferring causal relationships. Second, we lack data to compare individuals who declined to participate with those who did participate with respect to substance use and ART adherence which may impact our study findings. Third, ART adherence was measured by assessing past seven-day doses without asking the extent to which a missed dose on a given day was specifically related to substance use’s impact on non-adherence in a temporal, event-level nature. Similarly, we measured substance use in absolute terms (i.e., use versus no use), and did not have information about other aspects of substance use (e.g., frequency of use, intensity of use, mode of consumption, context of or reasons for use) so “substance use” and “polysubstance use” may have captured a range of frequency and intensity of use. More detailed assessment of use in future research would provide a more nuanced understanding of the relationship between substance use or polysubstance use and ART utilization using longitudinal study designs and real-time data collection such as ecological momentary assessment techniques. Fourth, self-reported measures are subject to social desirability and other biases, particularly with respect to substance use, leading participants to potentially underreport sensitive or socially undesirable behaviors. However, the anonymous nature of the survey aimed to minimize such potential biases. Fifth, with respect to cannabis use, we did not assess if current use was associated with recreational purposes or medical use (e.g., management of HIV-associated symptoms). While cannabis is the mostly commonly reported substance used among PWH [58], changing U.S. state-specific regulations related to cannabis will likely impact the ways researchers assess its use. Finally, our dataset lacks information on other social and structural determinants of health that may influence ART adherence such as housing instability, incarceration, food insecurity, stigmatization, racism, mental illness, and transportation barriers which may impact the substance use-ART adherence relationship and overall engagement in HIV care and treatment.

## 5. Conclusions

In summary, our study explored the relationship between substance use and ART adherence among a sample of racially and ethnically diverse PWH that allowed the examination of patterns by gender and smoking status, finding that substance use was commonly reported and associated with sub-optimal ART adherence. These findings underscore the adherence challenges among PWH who report substance use, which warrants appropriate nonjudgmental interventions such as screenings as part of routine clinical care [59] and the investment of resources into prevention strategies to improve health outcomes among PWH who report substance use. Our study adds to the growing literature on single and polysubstance use and the HIV care cascade, signaling targets of potential public health strategies, and suggests the prioritization of evidence-based, theory-informed interventions to improve adherence among PWH to support efforts in the Ending the HIV Epidemic initiative [1,60]. Please see Supplementary Materials section for our Citation Diversity Statement. 

## Figures and Tables

**Table 1:** HIV Clinical and Demographic Characteristics for the Full Sample and by Cigarette Smoking Status, Gender, and Antiretroviral Therapy (ART) Adherence.

Characteristic *	Full Sample *n* (%) or Median (IQR) *n* = 237	Smoking *n* (%) or Median (IQR) *n* = 112	Nonsmoking ^a^ *n* (%) or Median (IQR) *n* = 125	*p*-Value (Smoking vs. Nonsmoking)	Females ^b^ *n* (%) or Median (IQR) *n* = 100	Males *n* (%) or M +/− SD *n* = 137	*p*-Value (Female vs. Male)	ART Adherent *n* (%) or Median (IQR) *n* = 152	ART Nonadherent *n* (%) or Median (IQR) *n* = 85	*p*-Value (Adherent vs. Nonadherent)
**HIV clinical characteristics**										
Years since HIV diagnosis	21 (15–26)	22 (16–27)	21 (15–21)	0.82	21 (14–26)	21.5 (15–26)	0.12	21 (16–26)	21 (15–26)	0.76
AIDS diagnosis ^c^	108 (46.6)	57 (52.8)	50 (40.7)	0.07	56 (56.0)	52 (39.4)	0.01	64 (43.0)	42 (51.9)	0.20
**Demographic characteristics**										
Age	53 (45–58)	52 (45–57)	53 (45–59)	0.31	53 (41–58)	53 (46–58)	0.61	53 (46–58)	53 (41–58)	0.17
Race				0.26			0.64			0.12
White	24 (10.6)	13 (11.9)	11 (9.1)		10 (10.1)	14 (10.6)		20 (13.6)	4 (4.9)	
Black	125 (54.1)	63 (57.8)	61 (50.4)		57 (57.6)	68 (51.56		76 (51.7)	48 (58.5)	
Other ^d^	85 (35.5)	33 (30.3)	49 (40.5)		32 (32.3)9	50 (37.9)		51 (34.7)	30 (36.6)	
Education				0.57			0.51			0.01
1st–11th Grade	71 30.0)	37 (33.0)	35 (28.0)		27 (27.0)	44 (32.1)		42 (27.6)	29 (34.1)	
High School	34 (14.3)	14 (12.5)	20 (16.0)		14 (14.0)	20 (14.6)		29 (19.1)	5 (5.9)	
GED	31 (13.1)	17 (15.2)	14 (11.2)		17 (17.0)	14 (10.2)		14 (9.2)	16 (18.8)	
>High School	101 (42.6)	44 (39.3)	56 (44.8)		42 42.0)	59 (43.1)		67 (44.1)	35 (41.2)	
Sexual Orientation				0.04			<0.001			0.46
Heterosexual	175 (73.8)	79 (70.5)	96 (76.8)		57 (56.4)	119 (86.8)		115 (75.7)	59 (71.1)	
Homosexual	40 (16.9)	17 (15.2)	23 (18.4)		31 (30.7)	9 (6.6)		26 (17.1)	14 (16.9)	
Bisexual/Other	22 (9.3)	16 (14.3)	6 (4.8)		12 (12.9)	9 (6.6)		11 (7.2)	10 (12.1)	
Marital Status				0.75			0.04			0.78
Never Married	136 (57.4)	62 (55.4)	73 (58.4)		67 (67.0)	69 (50.4)		84 (55.3)	51 (60.0)	
Married	60 (25.3)	31 (27.7)	29 (23.2)		18 (18.0)	41 (29.9)		40 (26.3)	20 (23.5)	
Other ^e^	41 (17.3)	19 (17.0)	23 (18.4)		15 (15.0)	27 (19.7)		28 (18.4)	14 (16.5)	

Note: * Totals do not always sum to 100% due to missing data; IQR, interquartile range; bold indicates statistical significance (*p* < 0.05); ^a^ Including those who have never smoked cigarettes and those who smoked cigarettes in the past; ^b^ Including cisgender (*n* = 99) and transgender (*n* = 1) females; ^c^ Self-reported an AIDS diagnosis; ^d^ Included those who identified as American Indian/Alaskan Native, Asian, Native Hawaiian/Other Pacific Islander, or Other; ^e^ Included those who reported divorced, separated, widowed, or other.

**Table 2:** Association of Substance Use and Antiretroviral Therapy (ART). Non-Adherence among People with HIV (*n* = 227).

Correlate	Adjusted Odds Ratio	95% Confidence Interval	*p*-Value
One substance	**0.45**	0.23, 0.89	0.02
Polysubstance	**0.46**	0.22, 0.96	0.03
Gender	0.89	0.49, 1.60	0.71
Cigarette Smoking	3.94	1.33, 11.69	0.11

Note: Adjusted Odds Ratios (aORs) in bold indicate statistical significance at *p* < 0.05 when compared to the referent group of no substance use.

## Data Availability

Limited de-identified raw data available from the corresponding author upon reasonable request.

## Supplementary Materials

The following supporting information can be downloaded at: https://www.mdpi.com/article/10.3390/tropicalmed7110349/s1, File S1: Citation Diversity Statement [61,62,63,64,65,66,67,68,69,70,71,72].

## References

[1] Fauci A.S., Redfield R.R., Sigounas G., Weahkee M.D., Giroir B.P. (2019). Ending the HIV Epidemic: A Plan for the United States. JAMA.

[2] Centers for Disease Control and Prevention (2022). Monitoring Selected National HIV Prevention and Care Objectives by Using HIV Surveillance Data—United States and 6 Dependent Areas, 2020.

[3] Morne J.E., Tesoriero J.M., Martin E.G., Birkhead G.S., Holtgrave D.R., Hagos K., Zucker H. (2020). Ending the HIV Epidemic: New York’s Quest to Become the First State to Reduce HIV Prevalence. Public Health Rep..

[4] Ortego C., Huedo-Medina T.B., Llorca J., Sevilla L., Santos P., Rodríguez E., Warren M.R., Vejo J. (2011). Adherence to highly active antiretroviral therapy (HAART): A meta-analysis. AIDS Behav..

[5] Gardner E.M., McLees M.P., Steiner J.F., Del Rio C., Burman W.J. (2011). The spectrum of engagement in HIV care and its relevance to test-and-treat strategies for prevention of HIV infection. Clin. Infect. Dis..

[6] Okafor C.N., Zhou Z., Burrell L.E., Kelso N.E., Whitehead N.E., Harman J.S., Cook C.L., Cook R.L. (2017). Marijuana use and viral suppression in persons receiving medical care for HIV-infection. Am. J. Drug Alcohol Abus..

[7] Mimiaga M.J., Reisner S.L., Grasso C., Crane H.M., Safren S.A., Kitahata M.M., Schumacher J.E., Mathews W.C., Mayer K.H. (2013). Substance use among HIV-infected patients engaged in primary care in the United States: Findings from the Centers for AIDS Research Network of Integrated Clinical Systems cohort. Am. J. Public Health.

[8] SAMHSA (2021). Key Substance Use and Mental Health Indicators in the United States: Results from the 2020 National Survey on Drug Use and Health.

[9] Hartzler B., Dombrowski J.C., Crane H.M., Eron J.J., Geng E.H., Christopher Mathews W., Mayer K.H., Moore R.D., Mugavero M.J., Napravnik S. (2017). Prevalence and Predictors of Substance Use Disorders Among HIV Care Enrollees in the United States. AIDS Behav..

[10] Rosen M.I., Black A.C., Arnsten J.H., Goggin K., Remien R.H., Simoni J.M., Golin C.E., Bangsberg D.R., Liu H. (2013). Association between use of specific drugs and antiretroviral adherence: Findings from MACH 14. AIDS Behav..

[11] Mannheimer S., Hirsch-Moverman Y. (2015). What we know and what we do not know about factors associated with and interventions to promote antiretroviral adherence. Curr. Infect. Dis. Rep..

[12] Braithwaite R.S., Bryant K.J. (2010). Influence of alcohol consumption on adherence to and toxicity of antiretroviral therapy and survival. Alcohol Res. Health.

[13] Gonzalez A., Barinas J., O’Cleirigh C. (2011). Substance use: Impact on adherence and HIV medical treatment. Curr. HIV/AIDS Rep..

[14] Zhang Y., Wilson T.E., Adedimeji A., Merenstein D., Milam J., Cohen J., Cohen M., Golub E.T. (2018). The Impact of Substance Use on Adherence to Antiretroviral Therapy Among HIV-Infected Women in the United States. AIDS Behav..

[15] Mannes Z.L., Burrell L.E., Ferguson E.G., Zhou Z., Lu H., Somboonwit C., Cook R.L., Ennis N. (2018). The association of therapeutic versus recreational marijuana use and antiretroviral adherence among adults living with HIV in Florida. Patient Prefer. Adherence.

[16] Shuper P.A., Joharchi N., Irving H., Fletcher D., Kovacs C., Loutfy M., Walmsley S.L., Wong D.K., Rehm J. (2016). Differential predictors of ART adherence among HIV-monoinfected versus HIV/HCV-coinfected individuals. AIDS Care.

[17] Fulcher J.A., Javanbakht M., Shover C.L., Ragsdale A., Brookmeyer R., Shoptaw S., Gorbach P.M. (2021). Comparative impact of methamphetamine and other drug use on viral suppression among sexual minority men on antiretroviral therapy. Drug Alcohol Depend..

[18] Starks T.J., MacDonell K.K., Pennar A.L., Dinaj-Koci V., Millar B.M., Naar S. (2020). Drug Use Among Adolescents and Young Adults with Unsuppressed HIV Who Use Alcohol: Identifying Patterns of Comorbid Drug Use and Associations with Mental Health. AIDS Behav..

[19] Degarege A., Krupp K., Tamargo J., Martinez S.S., Campa A., Baum M. (2022). Polysubstance use and adherence to antiretroviral treatment in the Miami Adult Studies on HIV (MASH) cohort. AIDS Care.

[20] Parsons J.T., Starks T.J., Millar B.M., Boonrai K., Marcotte D. (2014). Patterns of substance use among HIV-positive adults over 50: Implications for treatment and medication adherence. Drug Alcohol Depend..

[21] Carter A., Roth E.A., Ding E., Milloy M.J., Kestler M., Jabbari S., Webster K., de Pokomandy A., Loutfy M., Kaida A. (2018). Substance Use, Violence, and Antiretroviral Adherence: A Latent Class Analysis of Women Living with HIV in Canada. AIDS Behav..

[22] Centers for Disease Control and Prevention (2021). Estimated HIV Incidence and Prevalence in the United States, 2015–2019.

[23] Maragh-Bass A.C., Gamble T., El-Sadr W.M., Hanscom B., Tolley E.E. (2021). Examining stigma, social support, and gender differences in unsuppressed HIV viral load among participants in HPTN 065. J. Behav. Med..

[24] Bomfim I.G.O., Santos S.S., Napoleão A.A. (2022). Adherence to Antiretroviral Therapy in People Living with HIV/AIDS: A Cross-Sectional Study. AIDS Patient Care STDS.

[25] Ortego C., Huedo-Medina T.B., Santos P., Rodríguez E., Sevilla L., Warren M., Llorca J. (2012). Sex differences in adherence to highly active antiretroviral therapy: A meta-analysis. AIDS Care.

[26] Matson T.E., McGinnis K.A., Rubinsky A.D., Frost M.C., Czarnogorski M., Bryant K.J., Edelman E.J., Satre D.D., Catz S.L., Bensley K.M. (2018). Gender and alcohol use: Influences on HIV care continuum in a national cohort of patients with HIV. AIDS.

[27] Shuter J., Bernstein S.L. (2008). Cigarette smoking is an independent predictor of nonadherence in HIV-infected individuals receiving highly active antiretroviral therapy. Nicotine Tob. Res..

[28] Webb M.S., Vanable P.A., Carey M.P., Blair D.C. (2009). Medication adherence in HIV-infected smokers: The mediating role of depressive symptoms. AIDS Educ. Prev..

[29] O’Cleirigh C., Valentine S.E., Pinkston M., Herman D., Bedoya C.A., Gordon J.R., Safren S.A. (2015). The unique challenges facing HIV-positive patients who smoke cigarettes: HIV viremia, ART adherence, engagement in HIV care, and concurrent substance use. AIDS Behav..

[30] Cornelius M.E., Loretan C.G., Wang T.W., Jamal A., Homa D.M. (2022). Tobacco Product Use Among Adults—United States, 2020. MMWR Morb. Mortal. Wkly. Rep..

[31] Weinberger A.H., Smith P.H., Funk A.P., Rabin S., Shuter J. (2017). Gender Differences in Tobacco Use among Persons Living with HIV/AIDS: A Systematic Review and Meta-Analysis. J. Acquir. Immune Defic. Syndr..

[32] Helleberg M., Afzal S., Kronborg G., Larsen C.S., Pedersen G., Pedersen C., Gerstoft J., Nordestgaard B.G., Obel N. (2013). Mortality attributable to smoking among HIV-1-infected individuals: A nationwide, population-based cohort study. Clin. Infect. Dis..

[33] Ale B.M., Amahowe F., Nganda M.M., Danwang C., Wakaba N.N., Almuwallad A., Ale F.B.G., Sanoussi A., Abdullahi S.H., Bigna J.J. (2021). Global burden of active smoking among people living with HIV on antiretroviral therapy: A systematic review and meta-analysis. Infect. Dis. Poverty.

[34] Centers for Disease Control and Prevention (2021). Considerations for Antiretroviral Use in Special Patient Populations.

[35] Weinberger A.H., Pang R.D., Seng E.K., Levin J., Esan H., Segal K.S., Shuter J. (2021). Self-control and smoking in a sample of adults living with HIV/AIDS: A cross-sectional survey. Addict. Behav..

[36] De Boni R.B., Shepherd B.E., Grinsztejn B., Cesar C., Cortés C., Padgett D., Gotuzzo E., Belaunzarán-Zamudio P.F., Rebeiro P.F., Duda S.N. (2016). Substance Use and Adherence Among People Living with HIV/AIDS Receiving cART in Latin America. AIDS Behav..

[37] Tsuyuki K., Shoptaw S.J., Ransome Y., Chau G., Rodriguez-Diaz C.E., Friedman R.K., Srithanaviboonchai K., Li S., Mimiaga M.J., Mayer K.H. (2019). The Longitudinal Effects of Non-injection Substance Use on Sustained HIV Viral Load Undetectability Among MSM and Heterosexual Men in Brazil and Thailand: The Role of ART Adherence and Depressive Symptoms (HPTN 063). AIDS Behav..

[38] Lesko C.R., Keruly J.C., Moore R.D., Shen N.M., Pytell J.D., Lau B., Fojo A.T., Mehta S.H., Kipke M., Baum M.K. (2022). COVID-19 and the HIV continuum in people living with HIV enrolled in Collaborating Consortium of Cohorts Producing NIDA Opportunities (C3PNO) cohorts. Drug Alcohol Depend..

[39] Lipira L., Rao D., Nevin P.E., Kemp C.G., Cohn S.E., Turan J.M., Simoni J.M., Andrasik M.P., French A.L., Unger J.M. (2020). Patterns of alcohol use and associated characteristics and HIV-related outcomes among a sample of African-American women living with HIV. Drug Alcohol Depend..

[40] Leddy A.M., Zakaras J.M., Shieh J., Conroy A.A., Ofotokun I., Tien P.C., Weiser S.D. (2021). Intersections of food insecurity, violence, poor mental health and substance use among US women living with and at risk for HIV: Evidence of a syndemic in need of attention. PLoS ONE.

[41] Vidrine D.J., Arduino R.C., Gritz E.R. (2007). The effects of smoking abstinence on symptom burden and quality of life among persons living with HIV/AIDS. AIDS Patient Care STDS.

[42] Mdodo R., Frazier E.L., Dube S.R., Mattson C.L., Sutton M.Y., Brooks J.T., Skarbinski J. (2015). Cigarette smoking prevalence among adults with HIV compared with the general adult population in the United States: Cross-sectional surveys. Ann. Intern. Med..

[43] Weinberger A.H., Funk A.P., Goodwin R.D. (2016). A review of epidemiologic research on smoking behavior among persons with alcohol and illicit substance use disorders. Prev. Med..

[44] Min J.Y., Levin J., Weinberger A.H. (2022). Associations of tobacco cigarette use and dependence with substance use disorder treatment completion by sex/gender and race/ethnicity. J. Subst. Abus. Treat..

[45] Weinberger A.H., Platt J., Esan H., Galea S., Erlich D., Goodwin R.D. (2017). Cigarette Smoking Is Associated With Increased Risk of Substance Use Disorder Relapse: A Nationally Representative, Prospective Longitudinal Investigation. J. Clin. Psychiatry.

[46] Azar P., Wood E., Nguyen P., Luma M., Montaner J., Kerr T., Milloy M.J. (2015). Drug use patterns associated with risk of non-adherence to antiretroviral therapy among HIV-positive illicit drug users in a Canadian setting: A longitudinal analysis. BMC Infect. Dis..

[47] Velloza J., Kemp C.G., Aunon F.M., Ramaiya M.K., Creegan E., Simoni J.M. (2020). Alcohol Use and Antiretroviral Therapy Non-Adherence Among Adults Living with HIV/AIDS in Sub-Saharan Africa: A Systematic Review and Meta-Analysis. AIDS Behav..

[48] Cherenack E.M., Enders K., Rupp B.M., Seña A.C., Psioda M. (2022). Daily Predictors of ART Adherence Among Young Men Living with HIV Who Have Sex with Men: A Longitudinal Daily Diary Study. AIDS Behav..

[49] Bahji A., Li Y., Vickers-Smith R., Crystal S., Kerns R.D., Gordon K.S., Macmadu A., Skanderson M., So-Armah K., Sung M.L. (2022). Self-Reported Cannabis Use and HIV Viral Control among Patients with HIV Engaged in Care: Results from a National Cohort Study. Int. J. Environ. Res. Public Health.

[50] Sheinfil A.Z., Foley J.D., Moskal D., Dalton M.R., Firkey M., Ramos J., Maisto S.A., Woolf-King S.E. (2022). Daily Associations Between Alcohol Consumption and Antiretroviral Therapy (ART) Adherence Among HIV-Positive Men Who Have Sex With Men. AIDS Behav..

[51] Kalichman S.C., Eaton L.A., Kalichman M.O. (2022). Believing That It Is Hazardous to Mix Alcohol With Medicines Predicts Intentional Nonadherence to Antiretrovirals. J. Acquir. Immune Defic. Syndr..

[52] Kalichman S.C., Eaton L.A., Kalichman M.O. (2022). Substance Use-Related Intentional Nonadherence to Antiretroviral Therapy Among Young Adults Living with HIV. AIDS Patient Care STDS.

[53] Chand S., DeMarino C., Gowen A., Cowen M., Al-Sharif S., Kashanchi F., Yelamanchili S.V. (2022). Methamphetamine Induces the Release of Proadhesive Extracellular Vesicles and Promotes Syncytia Formation: A Potential Role in HIV-1 Neuropathogenesis. Viruses.

[54] Wu K., Tie Y., Dasgupta S., Beer L., Marcus R. (2022). Injection and Non-Injection Drug Use Among Adults with Diagnosed HIV in the United States, 2015–2018. AIDS Behav..

[55] Miller W.C., Hoffman I.F., Hanscom B.S., Ha T.V., Dumchev K., Djoerban Z., Rose S.M., Latkin C.A., Metzger D.S., Lancaster K.E. (2018). A scalable, integrated intervention to engage people who inject drugs in HIV care and medication-assisted treatment (HPTN 074): A randomised, controlled phase 3 feasibility and efficacy study. Lancet.

[56] Bazzi A.R., Drainoni M.L., Biancarelli D.L., Hartman J.J., Mimiaga M.J., Mayer K.H., Biello K.B. (2019). Systematic review of HIV treatment adherence research among people who inject drugs in the United States and Canada: Evidence to inform pre-exposure prophylaxis (PrEP) adherence interventions. BMC Public Health.

[57] Lyons S.J., Dailey A.F., Yu C., Johnson A.S. (2021). Care Outcomes Among Black or African American Persons with Diagnosed HIV in Rural, Urban, and Metropolitan Statistical Areas—42 U.S. Jurisdictions, 2018. MMWR Morb. Mortal. Wkly. Rep..

[58] Pacek L.R., Towe S.L., Hobkirk A.L., Nash D., Goodwin R.D. (2018). Frequency of Cannabis Use and Medical Cannabis Use Among Persons Living With HIV in the United States: Findings From a Nationally Representative Sample. AIDS Educ. Prev..

[59] Panel on Antiretroviral Guidelines for Adults and Adolescents (2022). Guidelines for the Use of Antiretroviral Agents in Adults and Adolescents with HIV. https://clinicalinfo.hiv.gov/en/guidelines/hiv-clinical-guidelines-adult-and-adolescent-arv/whats-new-guidelines.

[60] Ameri M., Movahed E., Farokhzadian J. (2020). Effect of information, motivation, and behavioral skills model on adherence to medication, diet, and physical activity in HIV/ADIS patients: A health promotion strategy. J. Educ. Health Promot..

[61] Mitchell S.M., Lange S.S., Brus H. (2013). Gendered citation patterns in international relations journals. Int. Stud. Perspect..

[62] Maliniak D., Powers R.R., Walter B.F. (2013). The gender citation gap in international relations. Int. Organ..

[63] Caplar N., Tacchella S., Birrer S. (2017). Quantitative evaluation of gender bias in astronomical publications from citation counts. Nat. Astron..

[64] Dion M.L., Sumner J.L., Mitchell S.M. (2018). Gendered citation patterns across political science and social science methodology fields. Political Anal..

[65] Dworkin J.D., Linn K.A., Teich E.G., Zurn P., Shinohara R.T., Bassett D.S. (2020). The extent and drivers of gender imbalance in neuroscience reference lists. Nat. Neurosci..

[66] Bertolero M.A., Dworkin J.D., David S.U., Lloreda C.L., Srivastava P., Stiso J., Zhou D., Dzirasa K., Fair D.A., Kaczkurkin A.N. (2020). Racial and ethnic imbalance in neuroscience reference lists and intersections with gender. bioRxiv.

[67] Wang X., Dworkin J.D., Zhou D., Stiso J., Falk E.B., Bassett D.S., Zurn P., Lydon-Staley D.M. (2021). Gendered citation practices in the field of communication. Ann. Int. Commun..

[68] Chatterjee P., Werner R.M. (2021). Gender disparity in citations in high-impact journal articles. JAMA Netw. Open.

[69] Fulvio J.M., Akinnola I., Postle B.R. (2021). Gender (im)balance in citation practices in cognitive neuroscience. J. Cogn. Neurosci..

[70] Zhou D., Bertolero M.A., Stiso J., Cornblath E.J., Teich E.G., Blevins A.S., Virtualmario, Camp C., Dworkin J.D., Bassett D.S. Gender Diversity Statement and Code Notebook. v1.1. https://zenodo.org/record/4062888#.Y2PkvHbMJaQ.

[71] Ambekar A., Ward C., Mohammed J., Male S., Skiena S. Name-ethnicity classification from open sources. Proceedings of the 15th ACM SIGKDD international conference on Knowledge Discovery and Data Mining.

[72] Sood G., Laohaprapanon S. (2018). Predicting race and ethnicity from the sequence of characters in a name. arXiv.

