# Supplementary material for: Substance Use and Adherence to Antiretroviral Therapy among People Living with HIV in the United States
Source: Trop Med Infect Dis. 2022 Nov 4;7(11):349. doi: 10.3390/tropicalmed7110349 (PMC9697670; doi:10.3390/tropicalmed7110349)
Supplement: Supplementary file 1 [file tropicalmed-07-00349-s001.zip › tropicalmed-1974438-supplementary.pdf]

Tropical Medicine and Infectious Disease

Supplementary Materials for Substance Use and Adherence to Antiretroviral Therapy among People Living with HIV in the United States

Recent work in several fields of science has identified a bias in citation practices such that papers from women and other minority scholars are under-cited relative to the number of such papers in the field [61–69]. Here, we sought to proactively consider choosing references that reflect the diversity of the field in thought, form of contribution, gender, race, ethnicity, and other factors. First, we obtained the predicted gender of the first and last author of each reference by using databases that store the probability of a first name being carried by a woman [65,70]. By this measure (and excluding self-citations to the first and last authors of our current paper), our references contain 30.0% woman (first)/woman (last), 22.0% man/woman, 18.78% woman/man, and 29.22% man/man. This method is limited in that (a) names, pronouns, and social media profiles used to construct the databases may not, in every case, be indicative of gender identity and (b) it cannot account for intersex, non-binary, or transgender people. Second, we obtained predicted racial/ethnic category of the first and last author of each reference by databases that store the probability of a first and last name being carried by an author of color [71,72]. By this measure (and excluding self-citations), our references contain 8.24% author of color (first)/author of color (last), 13.61% white author/author of color, 24.27% author of color/white author, and 53.88% white author/white author. This method is limited in that (a) names and Florida Voter Data to make the predictions may not be indicative of racial/ethnic identity, and (b) it cannot account for Indigenous and mixed-race authors, or those who may face differential biases due to the ambiguous racialization or ethnicization of their names. We look forward to future work that could help us to better understand how to support equitable practices in science.

References

[61] Mitchell, S.M.; S. Lange, S.; Brus, H. Gendered citation patterns in international relations journals. *Int Stud Perspect* **2013**, 14, 485-492.

[62] Maliniak, D.; R. Powers, R.; Walter, B.F. The gender citation gap in international relations. *Int Organ* **2013**, 67, 889– 922.

[63] Caplar, N.; Tacchella, S.; Birrer, S. Quantitative evaluation of gender bias in astronomical publications from citation counts. *Nat Astron* **2017**, 1, 0141.

[64] Dion, M.L.; Sumner, J.L.; Mitchell, S.M. Gendered citation patterns across political science and social science methodology fields. *Polit Anal* **2018**, 26312–327.

[65] Dworkin, J.D.; Linn, K.A.; Teich, E.G.; Zurn, P.; Shinohara, R.T.; Bassett, D.S. The extent and drivers of gender imbalance in neuroscience reference lists. *Nat Neurosci* **2020**, 8, 918-926.

[66] Bertolero, M.A.; Dworkin, J.D.; David, S.U.; Lloreda, C.L.; Srivastava, P.; Stiso, J.; Zhou, D.; Dzirasa, K.; Fair, D.A.; Kaczkurkin, A.N.; Marlin, B.J.; Shohamy, D.; Uddin, L.Q.; Zurn, P.; Bassett, D.S. Racial and ethnic imbalance in neuroscience reference lists and intersections with gender. bioRxiv **2020**, 10.122020.10.12.336230.

[67] Wang, X.; Dworkin, J.D.; Zhou, D.; Stiso, J.; Falk, E.B.; Bassett, D.S.; Zurn, P.; Lydon-Staley, D.M. Gendered citation practices in the field of communication. *Ann Int Commun* **2021**, 45, 134.153.

[68] Chatterjee P.; Werner, R.M. Gender disparity in citations in high- impact journal articles. *JAMA Netw Open* **2021,** 4, e2114509.

[69] Fulvio, J.M.; Akinnola, I.; Postle, B.R. Gender (im)balance in citation practices in cognitive neuroscience. *J Cogn Neurosci* **2021**, 333–7.

[70] Zhou, D.; Bertolero, M.A.; Stiso, J.; Cornblath, E.J.; Teich, E.G.; Blevins, A.S.; Virtualmario; Camp, C.; Dworkin, J.D.; Bassett, D.S. Gender diversity statement and code notebook. v1.1. Available online: https://zenodo.org/record/4062888#.Y2PkvHbMJaQ (accessed on 28 September 2022).

[71] Ambekar, A.; Ward, C.; Mohammed, J.; Male, S.; Skiena, S. (2009, June). Name-ethnicity classification from open sources. In Proceedings of the 15th ACM SIGKDD international conference on Knowledge Discovery and Data Mining. Paris, France, (28 June 2009).
[72] Sood, G.; Laohaprapanon, S. Predicting race and ethnicity from the sequence of characters in a name. *arXiv preprint arXiv* **2018**, 1805, 02109.
